# Novel phage infecting the *Roseobacter* CHUG lineage reveals a diverse and globally distributed phage family

**DOI:** 10.1128/msphere.00458-24

**Published:** 2024-06-27

**Authors:** Zuqing Wu, Luyuan Guo, Ying Wu, Mingyu Yang, Sen Du, Jiabing Shao, Zefeng Zhang, Yanlin Zhao

**Affiliations:** 1 Fujian Provincial Key Laboratory of Agroecological Processing and Safety Monitoring, College of JunCao Sciences and Ecology, Fujian Agriculture and Forestry University, Fuzhou, China; University of Michigan, Ann Arbor, Michigan, USA

**Keywords:** *Roseobacter*, CHUG lineage, CHUG phages, genome sequence, ecological distribution

## Abstract

Bacteriophages play an essential role in shaping the diversity and metabolism of bacterial communities. Marine *Roseobacter* group is an abundant heterotrophic bacterial group that is involved in many major element cycles, especially carbon and sulfur. Members of the *Roseobacter* CHUG (Clade Hidden and Underappreciated Globally) lineage are globally distributed and are activated in pelagic marine environments. In this study, we isolated and characterized a phage, CRP-810, that infects the CHUG strain FZCC0198. The genome of CRP-810 was dissimilar to those of other known phages. Additionally, 251 uncultured viral genomes (UViGs) closely related to CRP-810 were obtained from the uncultivated marine viral contig databases. Comparative genomic and phylogenetic analyses revealed that CRP-810 and these related UViGs exhibited conserved genome synteny, representing a new phage family with at least eight subgroups. Most of the CRP-810-type phages contain an integrase gene, and CRP-810 can be integrated into the host genome. Further analysis revealed that three CRP-810-type members were prophages found in the genomes of marine SAR11, *Poseidonocella*, and *Sphingomonadaceae*. Finally, viromic read-mapping analysis showed that CRP-810-type phages were globally distributed and displayed distinct biogeographic patterns related to temperature and latitude. Many members with a lower G + C content were mainly distributed in the trade station, whereas members with a higher G + C content were mainly distributed in polar and westerlies station, indicating that the niche differentiation of phages was subject to host adaptation. Collectively, these findings identify a novel phage family and expand our understanding of phylogenetic diversity, evolution, and biogeography of marine phages.

**IMPORTANCE:**

The *Roseobacter* CHUG lineage, affiliated with the Pelagic *Roseobacter* Cluster (PRC), is widely distributed in the global oceans and is active in oligotrophic seawater. However, knowledge of the bacteriophages that infect CHUG members is limited. In this study, a CHUG phage, CRP-810, that infects the CHUG strain FZCC0198, was isolated and shown to have a novel genomic architecture. In addition, 251 uncultured viral genomes closely related to CRP-810 were recovered and included in the analyses. Phylogenomic analyses revealed that the CRP-810-type phages represent a new phage family containing at least eight genus-level subgroups. Members of this family were predicted to infect various marine bacteria. We also demonstrated that the CRP-810-type phages are widely distributed in global oceans and display distinct biogeographic patterns related to latitude. Collectively, this study provides important insights into the genomic organization, diversity, and ecology of a novel phage family that infect ecologically important bacteria in the global ocean.

## INTRODUCTION

Viruses are the most abundant and diverse biological entities found in marine environments. Bacteriophages infecting bacteria are the most dominant viruses in marine environments and outnumber bacteria by approximately 10-fold (1–3). Phages play vital roles in controlling bacterial mortality, shaping the structure and function of marine bacterial communities, affecting bacterial evolutionary processes, and altering biogeochemical cycles (4–8). Over the last decade, culture-independent metagenomic analyses have revealed tremendous genetic diversity and functional potential of marine phages (9–12). However, owing to the deficiency of cultivable bacterial hosts, the isolation and characterization of marine phages have progressed relatively slowly compared to culture-independent studies. Despite this, laboratory studies on phages infecting important bacterial lineages constantly emerged in recent years, such as pelagiphages (13–16), cyanophages (17–19), and roseophages (20–23). These studies illustrated the genomic features, evolutionary trajectory, infection strategy, and extensive biogeography distribution of some important marine phages, provided models for studying the host-phage interactions, and also helped to annotate the vast “virome dark matter” (24, 25).

The *Roseobacter* group of marine *Alphaproteobacteria* dominates coastal and polar environments and plays an important role in global carbon and sulfur cycling (26–28). The *Roseobacter* group is phylogenetically diverse and consists of at least 300 species and 100 distinct genus-level lineages (28). Among the diverse *Roseobacter* lineages, the CHAB-I-5, NAC11-7, DC5-80-3 (also called RCA), SAG-O19, ChesI-C, and CHUG lineages show a streamlined genome and oligotrophic environmental adaptation, forming a Pelagic *Roseobacter* Cluster (PRC) (29–31). Although PRC members account for the majority of roseobacters in the ocean (30, 31), they remain largely uncultivated and poorly studied (30). Unlike other PRCs, members of the *Roseobacter* CHUG lineage show no correlation with chlorophyll a (Chl-a) and phytoplankton abundance and lack two essential genes (*cobG* and *cbix*) involved in *de novo* vitamin B12 (VB12) synthesis (31, 32). In addition, CHUG strains can utilize L-fucose as the sole carbon source whereas all other PRC members lack the key gene, alpha-L-fucosidase gene (*fucA*) to utilize L-fucose. These evidences suggest that the CHUG cluster may have evolved a different niche from the other *Roseobacter* groups (31, 32).

Currently, more than 64 phages that infect diverse *Roseobacter* lineages have been reported (20, 22, 23, 33–38). Comparative genomic analysis classified the isolated roseophages into 16 groups. Although most roseophages were isolated on copiotrophic roseobacters that could proliferate on nutrient-rich plates (33–36), some roseophages infecting the oligotrophic PRC members have recently been reported (20, 22, 23, 37, 38). These PRC phages form six clades and are diverse, abundant, and widely distributed in oceans (20, 22, 23, 37, 38). Among them, one roseophage infecting CHUG member has been recently isolated and represents the first roseophage of the family *Autographiviridae* (38).

Here, the CHUG strain, FZCC0198, was used as the host for phage isolation. One CHUG phage, CRP-810, was isolated, and 251 uncultured viral genomes (UViGs) closely related to CRP-810 were identified. Analyses of the genomic diversity, evolutionary relationships, and taxonomic classification of these phages revealed that they represent a new family in the *Caudoviricetes* class. Furthermore, the distribution patterns of these phages were analyzed using viromic read-mapping analysis. Our results show that CHUG phages have genomic characteristics distinct from those of other known phages, representing a novel, diverse, and globally distributed phage family.

## RESULTS AND DISCUSSION

### Host strain

The 16S rRNA gene sequence of host FZCC0198 exhibited a high level of similarity with the 16S rRNA gene sequence of the CHUG strain HKCCA1288 (99.85%) (31). Phylogenetic analysis based on the 16S rRNA gene sequences also showed that FZCC0198 was placed on a branch of the CHUG lineage represented by HKCCA1288, indicating that it belongs to the CHUG lineage (Fig. S1).

### Biological and genomic characteristics of CRP-810

**Fig 1:**
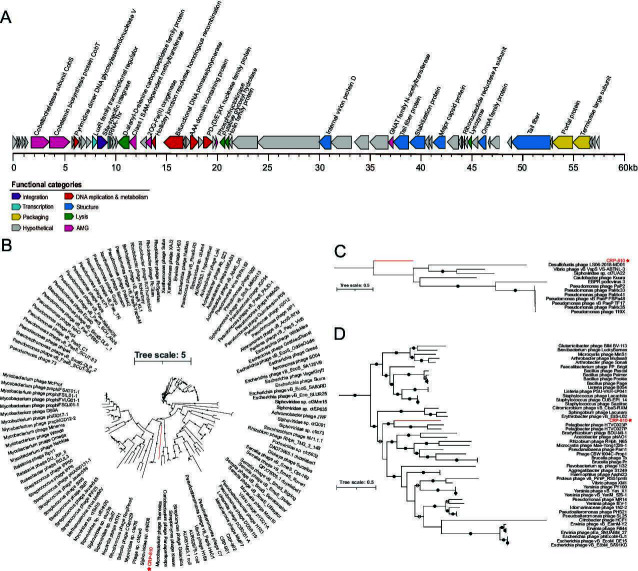
Genomic characterization and phylogenetic analysis of CRP-810. (**A**) Genome arrangement of CRP-810. The ORFs are indicated as arrows and color-coded according to their putative functions. (**B**) Phylogenetic tree of the bifunctional DNA primase/polymerase gene of CRP-810. (**C**) Phylogenetic tree of the capsid of CRP-810. (**D**) Phylogenetic tree of the terminase large subunit of CRP-810. CRP-810 is indicated with red asterisk.

CRP-810, which infects the CHUG strain FZCC0198, was isolated from the coastal surface water of Aoshan Bay, Yellow Sea, China in May 2022. The genome size of CRP-810 was 57.7 kb, encoding 63 open reading frames (ORFs). The G + C content of CRP-810 was 52.85%, which is similar to that of its host (56.13%). One Thr-tRNA gene was found in CRP-810. Genomic annotation showed that CRP-810 has limited similarity with other known phages in terms of genome content and arrangement. Approximately 41% of all the predicted ORFs in CRP-810 were assigned putative biological functions based on their homologs identified in GenBank or conserved domain analysis (Table S1). The annotated genes were mainly associated with DNA replication and metabolism, viral structure, DNA packaging, and lysis (Fig. 1A).

Four genes related to DNA replication and metabolism, including those encoding AAA family ATPase (ORF26, PF13479), bifunctional DNA primase-polymerase (ORF23), PD-(D/E)XK nuclease family protein (ORF28), and Holliday junction resolvase (ORF22), were identified in CRP-810 (Fig. 1A; see also Table S1). The gene encoding the AAA family ATPase has been found in some phages and was hypothesized to be involved in phage genome replication and energy provision (39, 40). The bifunctional DNA primase-polymerase in CRP-810 has an N-terminal bifunctional DNA primase-polymerase domain (bifunctional Prim-Pol, PF09250) and an AAA_25 domain (PF13481.9), suggesting that it may harbor both primase and polymerase activities (41). The phylogenetic tree of the Prim-Pol domain showed that CRP-810 formed a branch distinct from other known phages (Fig. 1B). The PD-(D/E)XK nuclease family protein in CRP-810 contains a YqaJ-like viral recombinase domain (PF09588) and shares 33.46% amino acid identity with that of roseophage CRP-902 (37). The YqaJ protein can digest linear double-stranded DNA and serves as part of the two-component SynExo viral recombinase functional unit; therefore, it may act as an endonuclease (42). In addition, we found that ORF9 encodes a pyrimidine dimer DNA glycosylase/endonuclease V (PDG) gene, which has both DNA glycosylase and APlyase activities, and can remove pyrimidine dimers, repair damaged DNA, and increase sensitivity to UV radiation (43, 44).

In the structural and packaging module, genes encoding the capsid protein (ORF45), stabilization protein (ORF42), internal virion protein D (ORF36), tail fiber protein (ORF57), portal protein (ORF59), and terminase large subunit (TerL) (ORF60) were identified (Fig. 1A; see also Table S1). The tail fiber in CRP-810 shared 48.80% amino acid identity with that of the pelagiphage HTVC025P (45). The capsid in CRP-810 genomes was most similar to that of *Vibrio* phage vB_VspS_VS-ABTNL-3 (30.94% amino acid identity). The phylogenetic tree of the capsid protein indicated that CRP-810 formed a separate branch (Fig. 1C). The phage portal protein is a channel through which DNA enters the virion during packaging and exits during ejection (46). The portal gene in CRP-810 shared weak similarity with that of the *Vibrio* phage *Phriendly* (25.76% amino acid identity). The TerL in CRP-810 shared 34.21% and 34.30% amino acid identity with those in HTVC023P and HTVC027P, respectively. Furthermore, the TerL phylogenetic tree showed that CRP-810 is located at the nearby branch of pelagiphages HTVC023P and HTVC027P (Fig. 1D). Overall, these results suggest that CRP-810 is a novel phage.

### CRP-810-type phages represent a novel and diverse family in *Caudoviricetes* class

To expand the diversity of the CRP-810-type phages, 317 UViGs were identified from the uncultivated viral contig databases, and 251 nonredundant UViGs were retained for further analyses. The genome size of the retrieved UViGs with nearly-complete genome (≥95% genome completeness) varied from 52.2 kb to 67.8 kb, and their G + C content ranged from 35.01% to 65.57% (Table S2). Furthermore, a total of 1,328 orthologous protein groups (≥2 members) were identified, among which 129 proteins were assigned putative biological function, and 16 genes were identified as core genes including capsid, bifunctional Prim-Pol, TerL, portal protein, AAA family ATPase, and PD-(D/E)XK nuclease (Table S3).

**Fig 2:**
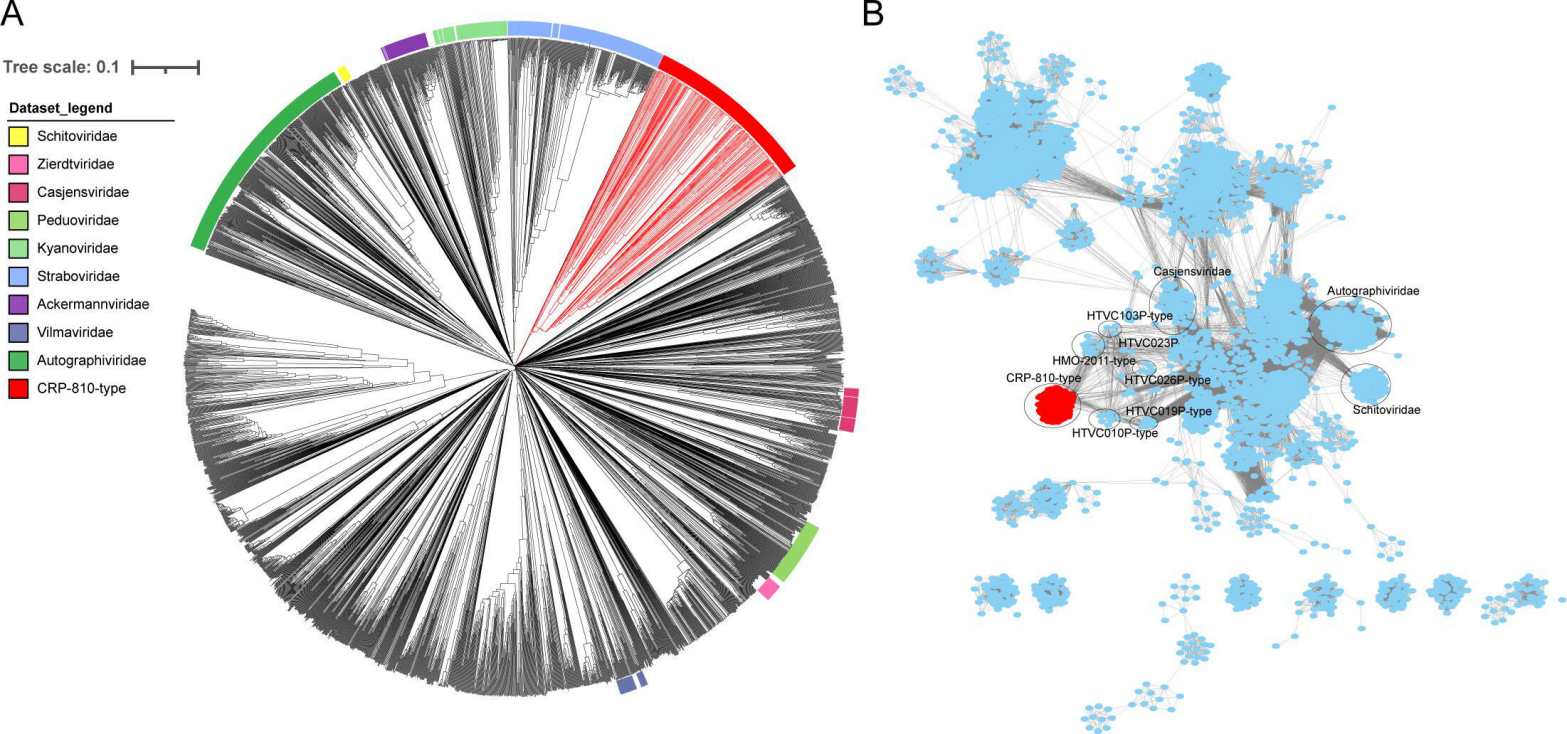
Evidence supporting the taxonomy of CRP-810-type phages. (**A**) Genome-wide proteomic tree for CRP-810, related UViGs, and other known prokaryotic dsDNA phages. The CRP-810-type phages were colored red. (**B**) Gene content-based viral network of CRP-810, virus from the NCBI-Ref database, and related UViGs.

**Fig 3:**
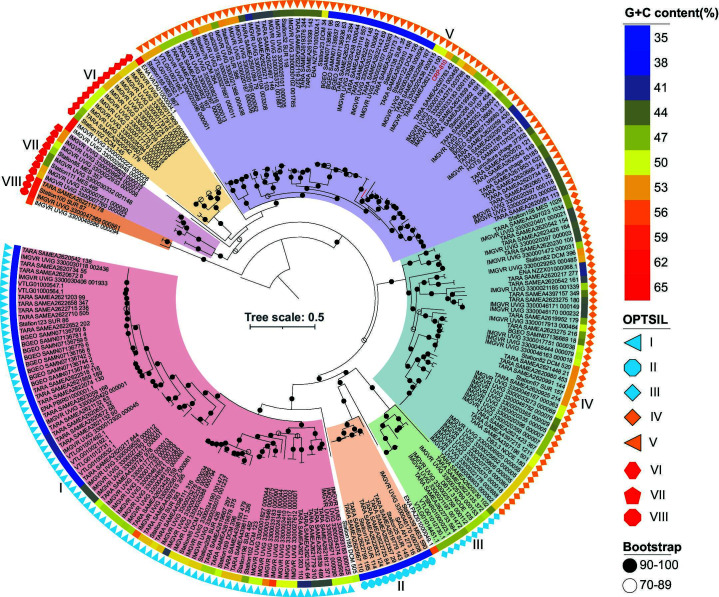
Phylogenic tree of CRP-810-type phages based on the amino acid sequences of six core genes. CRP-810-type phages were grouped into eight subgroups and six singletons marked by different colors. The G + C content and the result of OPTSIL are indicated in different colors and shapes. Solid and hollow circles in the phylogeny indicate nodes with bootstrap values >90% and >70%, respectively.

To elucidate the genetic diversity and taxonomy of CRP-810-type phages, various phylogenetic analysis tools based on amino acid sequences were performed. The proteomic tree generated by ViPTree showed that CRP-810-type phages formed a separate branch from other known phages (Fig. 2A). The protein-sharing networks also showed a significant distance between the CRP-810-type phages and other known viruses. They were only distantly related to the HMO-2011-type, HTVC103P-type, and HTVC010P-type pelagiphages (Fig. 2B; Table S4). The OPTSIL taxon prediction based on ViPTree suggested that the CRP-810-type phages can be classified into a new phage family with eight subgroups (I to VIII). The core gene-based phylogenomic tree also revealed that the CRP-810-type phages can be separated into eight subgroups (Fig. 3). Subgroups I, IV, and V comprised 80, 49, and 73 members, respectively. Others possess fewer members. CHUG phage CRP-810 was grouped into subgroup V. The AAI (average amino acid identity) and shared gene analyses showed that most of the AAI values and percentages of the shared gene between genomes within the same subgroup were >60% and >50%, respectively (Fig. S2). Overall, the above evidence suggests that CRP-810 and the CRP-810-type UViGs represent a novel phage family with at least eight subgroups in the *Caudoviricetes* class.

### Conserved genomic structure and variations in the CRP-810-type phages

**Fig 4:**
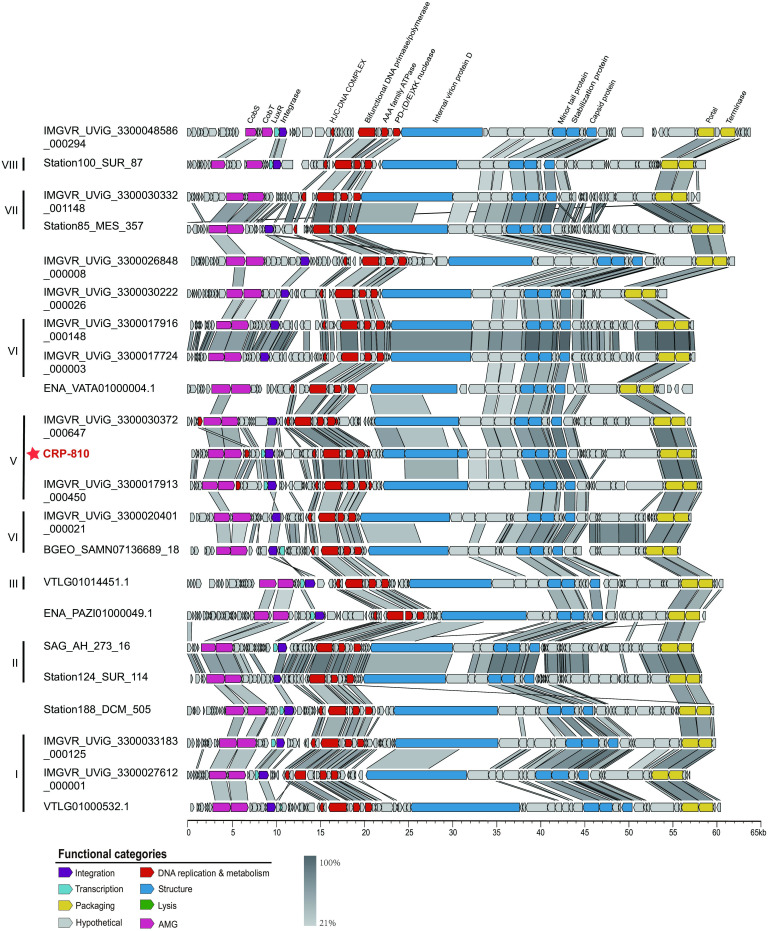
Alignment and comparison of genomes of the CRP-810-type phages from the major subgroups. CRP-810 is indicated by red asterisks. Predicted ORFs are represented by arrows, with the left or right arrow points indicating the direction of their transcription. ORFs annotated with known functions are marked using distinct colors according to their functions.

Genome comparison showed that all the CRP-810-type UViGs shared conserved genome synteny with CRP-810, and their genomes could also be roughly divided into DNA replication and metabolism module, morphology module, and packaging module (Fig. 4). In the DNA replication and metabolism module, almost all the CRP-810-type phages possessed genes encoding AAA domain-containing protein, PD-(D/E)XK nuclease family protein, holliday junction resolvase and single-stranded DNA annealing protein (Fig. 4; see also Fig. S3). We also found that some genes were exclusively present in certain subgroups. For example, the nucleotide modification associated domain, which may be involved in DNA or RNA modification was mainly identified in subgroup V. Another DNA modification gene, DNA methyltransferase, was sporadically distributed in subgroups I, III, IV, and V (Fig. S3). In the morphology and packaging module, several genes, including those encoding tail fiber protein, minor tail protein, internal virion protein D, TerL, and portal protein, were shared by all subgroups, suggesting that CRP-810-type phages have similar morphological and packaging characteristics (Fig. 4; see also Fig. S3). The tail assembly chaperone protein, which plays an important role in the maturation of bacteriophage tail fibers, was mainly annotated in subgroups I and IV (Fig. 4). Genes encoding baseplate-related proteins were also identified in subgroups II, IV, V, and VII (Fig. 4).

AMGs (auxiliary metabolic genes) are presumed to originate from the host and play roles in the regulation of host metabolism during host infection, therefore benefiting phage production (47–49). Nine AMGs were identified in the CRP-810-type phages. Almost all the CRP-810-type phages possessed genes encoding two cobalt chelatase subunits (*cobS* and *cobT*) (Fig. 4; see also Fig. S3). The product of the *cobS* gene is predicted to catalyze the last step of bacterial VB12 synthesis, and also found in several cyanophages and an archaeal virus (50–52). However, the function of *cobS* genes in phages remains uncertain (51). The *cobT* gene, which activates the lower ligand base for attachment to the nucleotide loop during cobamide biosynthesis (53), was also present in the CRP-810-type phages and clustered with *cobS*.

Another gene encoding a LuxR family transcriptional regulator (LuxR) was predicted in CRP-810 and 107 CRP-810-type UViGs. LuxR is involved in the quorum sensing in bacteria and is found in diverse bacteriophages, such as *flavobacterial* phages, *Iodobacteria* phages, and *Vibrio* phages (54–56). The LuxR may be responsible for the density-dependent transcriptional regulation of bacterial populations and for controlling phage lytic or lysogenic cycles (57–60). Therefore, CRP-810-type phages may switch from lysogeny to the lytic cycle by regulating the expression of LuxR, although further evidence is required to elucidate the function of this phage-encoded LuxR.

The GNAT family N-acetyltransferases (GNATs) were found in several subgroups, including subgroups I, IV, V, VI, and VII (Fig. 4; see also Fig. S3). Previous studies have shown that GNATs can perform diverse cellular functions, including the carbohydrates metabolism, energy metabolism, nucleotide metabolism, and stress regulation (61). Moreover, conserved 2OG-Fe(II) oxygenases, which are involved in DNA repair (18), were exclusively identified in subgroups III, IV, VI, and VII (Fig. 4; see also Fig. S3). The remaining four AMGs were genes encoding glycoproteins, thioredoxin, cyclopropane fatty acyl phospholipid synthase, and aspartyl hydroxylase. They were only predicted in subgroup V or VII (Fig. 4; see also Fig. S3 and Table S3). The glycoprotein was identified as glycogen synthase and involved in carbohydrate metabolism (62) and the thioredoxin serves as a hydrogen donor to ribonucleotide reductase (62, 63).

### CRP-810-type phages have a lysogenic life cycle and infect a variety of bacterial hosts

**Fig 5:**
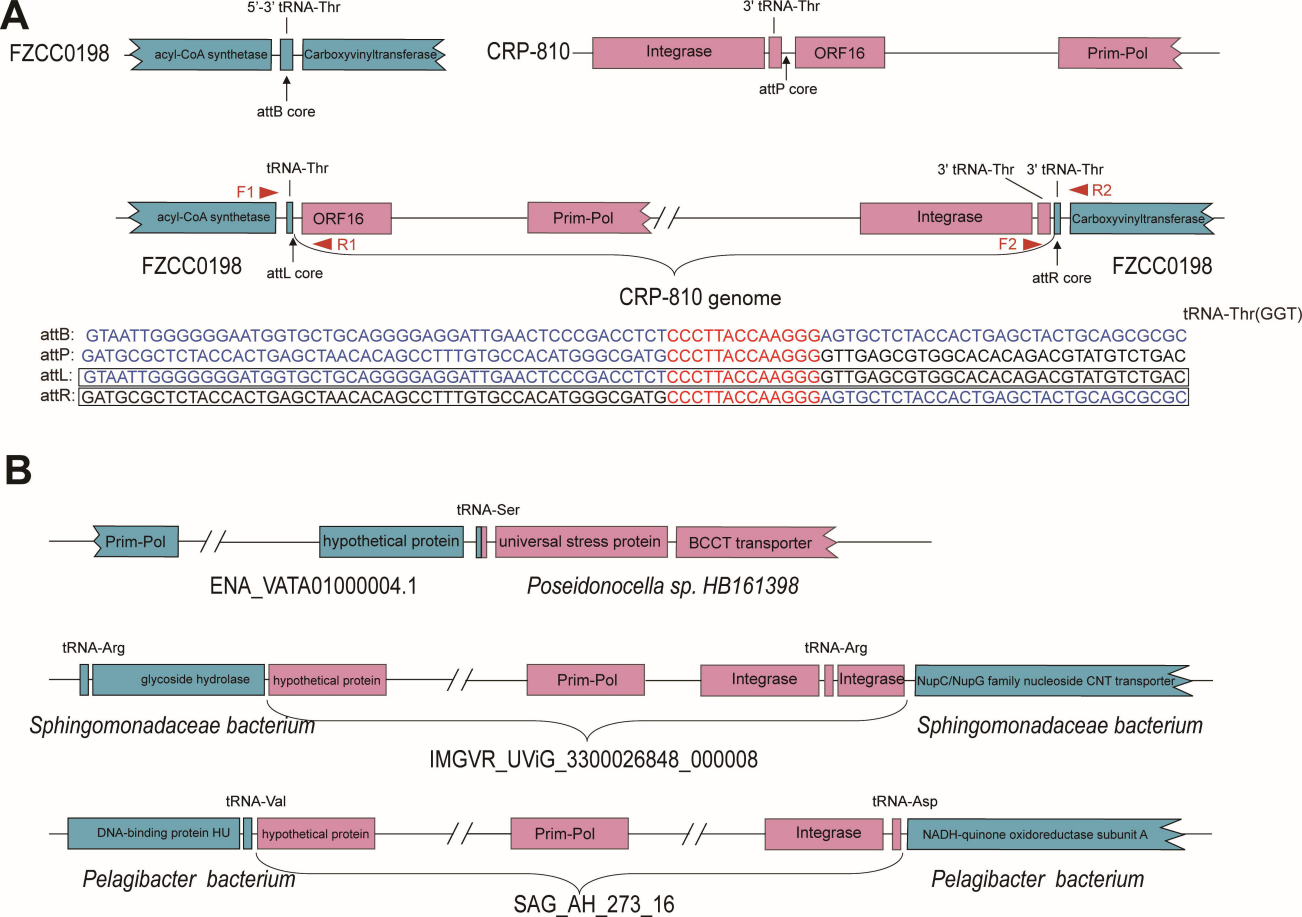
The verification and identification of CRP-810-type prophages. (**A**) Gene map and alignment of the DNA sequences of CRP-810 integration sites. (**B**) CRP-810-type prophages identified from the uncultured marine bacteria genomes database. Host and phage genes are shown in blue and pink, respectively.

Phage-encoded integrases, which are responsible for the site-specific integration and excision of phage genomes into hosts, are generally regarded as the hallmark genes of phage lysogenic life strategy (64). CHUG phage CRP-810 and most of the CRP-810-type UViGs (217 of 251 UViGs) encoded a site-specific integrase gene, suggesting that most CRP-810-type phages may have a lysogenic life cycle (Fig. 4; see also Fig. S3). To identify whether CRP-810 has a lysogenic life cycle, we then searched for attachment sites (*attP* and *attB*) in the genomes of CRP-810 and the host FZCC0198. A 13-bp identical sequence was found in the middle of the host tRNA-Thr gene and downstream of the phage tRNA-Thr gene (Fig. 5A). PCR amplification of the attachment sites *attL* and *attR* verified that CRP-810 can be integrated into the tRNA-Thr site in the host genome (Fig. 5A; see also Fig. S4).

Among the CRP-810-type UViGs, three were prophage sequences that reside within the bacterial genomes. The UViGs SAG_AH_273_16 (subgroup II), ENA_VATA01000004.1 (singleton III), and IMGVR_UViG_3300026848_000008 (singleton V) were identified as prophage that may infect SAR11, *Poseidonocella* sp*. HB161398*, and *Sphingomonadaceae* bacterium, respectively (Fig. 5B). The RaFAH prediction software based on protein content was used to predict potential hosts of the CRP-810-type phages. The results indicated that CRP-810-type phages may infect different bacterial groups based on a score cutoff of 0.5, including *Sphingomonadaceae, Roseobacter,* and SAR11 (Table S5). The potential host of subgroup II was predicted as SAR11, and subgroup II had an average G + C content of 35.60%. In summary, these results suggest that CRP-810-type phages can infect different hosts, although more evidence is required to confirm this using isolated phages.

### Ecological distribution of CRP-810-type phages

**Fig 6:**
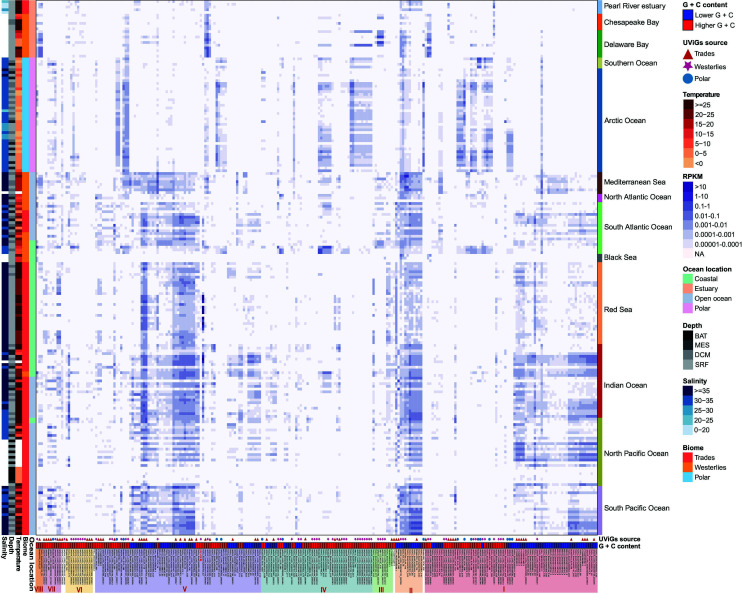
Biogeographical distribution of CRP-810-type phages in the global oceans. The relative abundance of phages was normalized as the RPKM. The colored bars on the left show the salinity, depth, temperature, marine biome and oceanic regions of the stations. The colored bar on the bottom depicts the G + C content of the phages, with higher G + C content (>45%) shown in red and lower G + C content shown in blue (<45%). The UViGs recovered from tropical, temperate, and polar regions are indicated with triangles, asterisks, and circles, respectively.

To assess the biogeographical distribution of CRP-810-type phages, we performed a viromic read-mapping analysis. The results showed that CRP-810-type phages can be detected globally, from coastal to pelagic regions, as well as from tropical to polar regions. We found that phages within the same subgroup displayed different distribution (Fig. 6). CRP-810 displayed a wide distribution as its CHUG host, being detected in 19 marine viromes (Fig. 6). 232 of 251 CRP-810-type UViGs also showed a wide distribution, being detected in at least 10 marine viromes (Fig. S5). Some CRP-810-type phages were detected more frequently at the polar and westerlies stations than at the trade stations (Fig. 6; see also Fig. S5), suggesting that their hosts may have adapted to cold oceanic regions. These CRP-810-type phages mostly originated from polar and westerlies samples and showed relatively higher G + C content (>45%). Linear regression analysis showed that most of their RPKM values displayed negative correlations with temperature and positive correlations with latitude (Table S6). In contrast, some lower G + C (<45%) CRP-810-type phages assembled from the trade samples were more prevalent in the trade than in westerlies and polar regions, and most of their RPKM values were positively correlated with temperature and negatively correlated with latitude (Table S6). These lower G + C phages were also more prevalent in open ocean and coastal regions, but were rarely detected in estuaries (Fig. 6). Overall, the lower G + C CRP-810-type phages displayed higher RPKM values than higher G + C CRP-810-type phages (*P* value <0.01, Mann–Whitney *U*-tests; Fig. S6). In addition, some higher G + C phages in subgroups I, III, V, and VIII were mainly detected in the estuaries with lower salinity and were significantly negatively correlated with salinity (Fig. 6; see also Table S6), suggesting that their hosts may inhabit estuarine environments.

### Conclusion

Here, we reported a novel phage infecting marine *Roseobacter* CHUG lineage and identified 251 related UViGs from the marine metagenomic datasets. We showed that CRP-810-type phages represent a novel phage family with eight subgroups. Our study also revealed that CRP-810-type phages are highly diverse and prevalent in marine environments. We discovered a novel and ecologically significant phage family and expanded the current knowledge regarding the diversity and evolution of marine roseophage. The CHUG phage we isolated provides a unique model system for studying marine virus-host interactions.

## MATERIALS AND METHODS

### Cultivation of host strains and amplification of 16S rRNA gene sequence

The host strain, FZCC0198, was isolated in September 2021 from the coastal water of Aoshan Bay, Yellow Sea, China (36.61°N, 121.16°E) using the dilution-to-extinction cultivation method (65, 66), and the FZCC0198 was purified at least three times using dilution-to-extinction cultivation method (65, 66). This purified strain was grown in dark at 26°C with a seawater-based growth medium containing a vitamin mixture and supplemented with 1 mM NH_4_Cl, 100 mM KH_2_PO_4_, 1 nM FeCl_3_, and a mixed carbon source (65, 66). The primers 16S-27F and 16S-1492R were used to amplify the 16S rRNA gene of FZCC0198. The 16S rRNA gene sequence of FZCC0198 was obtained by Sanger sequencing and assembled using ChromasPro (Technelysium Pty. Ltd., Tewantin, QLD, Australia).

### CHUG phage isolation

The seawater sample used for phage isolation was collected from the coastal water of Aoshan Bay, Yellow Sea, China (36.61°N, 121.16°E). The collected seawater was filtered through a 0.1-μm pore-size filter to remove cellular particles and stored at 4°C before being used for phage isolation. The phage was isolated using the liquid method of incubation with the host as previously reported in the literature (13). Briefly, the filtered seawater was inoculated into FZCC0198 cultures (10^4^ cells/mL). The growth of the FZCC0198 cultures was monitored using the Guava easyCyte flow cytometer (Merck Millipore, USA). When a decrease in bacterial density of FZCC0198 cells was observed, the lysed cultures were collected and filtered with 0.1 μm pore-size filters. The filtered lysates were stored at 4°C. Effort was performed to purify the phage using the dilution-to-extinction method (65, 66).

### Phage DNA preparation and high throughput sequencing of phage DNA

The filtered phage lysate was concentrated using Amicon Ultra centrifugal filters (30 kDa cutoff). The phage DNA was extracted using a DNeasy Blood & Tissue kit. The complete genome of the CHUG phage was sequenced using the Illumina paired-end HiSeq 2500 sequencing approach (2 × 150 bp) at Novogene (Beijing, China). Adapter sequences and low-quality reads were removed using Fastp v0.23.2 (67). *De novo* assembly of phage genome was performed using MEGAHIT v1.2.9 with default settings (68).

### Metagenomic retrieval of the CRP-810-type UViGs

Metagenomic viral and bacterial genomes from IMG/VR database v4 (69), station ALOHA assembly-free virus genomes (70), and metagenomic assembly of bacterial genome datasets (71, 72), as well as SAGs from the coastal Gulf of Maine (73), were downloaded for this analysis. The amino acid sequences of hallmark genes of CRP-810, including bifunctional DNA primase-polymerase, phage capsid, and terminase large subunit (TerL), were employed to create Profile hidden Markov models (HMMs) using hmmbuild command of HMMER (74). The HMM profiles were used to query the downloaded UViGs using the hmmsearch program of HMMER (e-value ≤10^−3^; bitscore ≥50) (74). Only matches with 25% amino acid identity and at least 80% alignment coverage were considered. Check V v1.0.1 (75) and CD-HIT v4.8.1 (76) were used to eliminate any low-quality (genome completeness <50%) and repetitive sequences (identity >0.95). The above analysis resulted in obtaining 251 UViGs for further analysis.

### Genome annotation and comparative genomic analysis

Putative ORFs of the CRP-810-type phages were predicted by GeneMarks (77), Prodigal v2.6.3 (78), and manual inspection. The functional annotation of the translated ORFs was performed using BLASTp against the NCBI nonredundant (nr) and the NCBI-RefSeq databases (amino acid identity ≥25%, alignment coverage ≥50%, and e-value <10^−3^). PFAM database was used to identify protein families and conserved domains (79). HHpred server was used to identify distant protein homologs (80). The orthologous protein groups of CRP-810-type genomes were detected using OrthoFinder v2.5.4 (81) based on sequence similarity (BLASTp option: e-value ≤10^−3^; ≥25% identity; ≥50% alignment coverage). tRNAs were detected using tRNAscan-SE v2.0.11 (82). The AAI values of pairwise comparisons was calculated using EzAAI (83). Finally, representative CRP-810-type genomes were visualized using EasyFig v2.2.2 (84).

### Phylogenetic analysis

Six core genes, including portal protein, terminase large subunit, major capsid protein, PD-(D/E)XK nuclease family protein, AAA family ATPase, and bifunctional DNA primase-polymerase were used to deduce the evolutionary relationship of the CRP-810-type phages. Briefly, the amino acid sequences of the core genes were aligned using MAFFT (85) and edited using TrimAl v1.4.1 (86). The alignments were concatenated using perl script, and the phylogenetic tree was constructed using IQ-TREE v2.2.0.3 (87). In addition, the taxonomic position of the CRP-810-type phages was verified using ViPTree (https://www.genome.jp/viptree/) (88). Phylogenetic trees were visualized and annotated using the Interactive Tree of Life (iTOL) v5 tool (89).

### Network analysis

The NCBI-RefSeq v217 database was used as the reference database for network analysis, including 12,044 phage genomes with 636,258 proteins. All the proteins were compared using all-versus-all BLASTp (e-value <10^−5^, bitscore ≥50), and the protein clusters were defined using the Markov clustering algorithm. vConTACT2 v0.11.3 (90) was used to calculate the similarities scores between the phage genomes and define the virus cluster. The network was visualized using Cytoscape v3.5.1 with an edge-weighted spring-embedded model.

### Determination of the phage integration sites and potential prophage

The integration sites of CRP-810 were identified using a method described in a previous study (45). Briefly, raw reads of phage sequencing were quality-filtered, trimmed, and mapped to the CRP-810 genome using CLC Genomic Workbench 11.0.1. Mapped sequences were manually inspected to detect the phage-host junctions. The resulting phage-host hybrid sequences were analyzed to identify the integration sites and their locations on the host genomes. PCR primer sets (attL-F, CCAACAAACTCGTCGCCTTC and attL-R, GCCATGATCCATAGCGCAGA; attR-F, ATAAGAGGGCCGCACACATC and attR-R, AGGATTTCCGTGCGCATGAT) were designed based on the predicted integration sites. DNA extracted from CRP-810-infected host cells was used as the PCR template.

Furthermore, to identify the putative CRP-810-type prophages, CRP-810-type phages were used as queries to search against metagenome-assembled genomes (72) using BLASTn (e-value <10^−3^, coverage ≥50%, identity ≥95%). Retrieved sequences from the metagenome-assembled genomes were annotated with the putative biological functions described above. The phage sequences were manually removed based on the results of the gene annotation. Finally, the remaining sequences were compared with known bacterial sequences using BLASTp and BLASTn against the NCBI nonredundant (nr) and the NCBI-RefSeq databases.

### Host prediction

The potential hosts of CRP-810-type UViGs were predicted using the RaFAH tool with default settings (91). The training and validating random forest model for RaFAH was built with 4,451 host-known phages, including CRP-810, CRP-810-type prophages, and 4,451 bacteriophages downloaded from NCBI RefSeq (v215).

### Ecological distribution

The relative abundance of the CRP-810-type phages in global marine viromes was estimated through a read-mapping analysis. Global Oceans Viromes (9, 92), Pearl River estuary virome (93), Mariana Trench virome (94), Eastern Tropical North Pacific virome (12), the Chesapeake Bay and Delaware Bay viromes (95), Black Sea virome (96), Red Sea virome (97), and South China Sea DNA virome (98) were downloaded to access the relative abundance of the phages. Viromic reads were mapped against the nonredundant set of CRP-810-type phage genomes (cd-hit -c 0.95 -aS 0.8) using CoverM v0.6.1 (https://github.com/wwood/CoverM) (≥95% nucleotide identity, ≥50 min read aligned length, and ≥80% read coverage). The relative abundance of CRP-810-type phages was normalized by RPKM. Differences in the abundance of the phages were plotted using the pheatmap and ggplot2 R package.

## Data Availability

The complete phage genome of CRP-810 has been deposited in the GenBank database under the accession number OR671924. The sequence of the 16S rRNA gene of FZCC0198 has been deposited in the NCBI GenBank database under accession number PP816027.

## SUPPLEMENTAL MATERIAL

Supplemental figures msphere.00458-24-s0001.docx Fig. S1 to S6

Table S1 msphere.00458-24-s0002.xlsx Genome annotation of CRP-810.

Table S2 msphere.00458-24-s0003.xlsx General features of the CRP-810-type genomes.

Table S3 msphere.00458-24-s0004.xlsx List of protein orthologous groups identified from the CRP-810-type genomes.

Table S4 msphere.00458-24-s0005.xlsx Distance between CRP-810-type phages and other related known phages based on the gene contents.

Table S5 msphere.00458-24-s0006.xlsx Potential host of the CRP-810-type phages predicted by Rafah.

Table S6 msphere.00458-24-s0007.xlsx Correlation between the CRP-810-type phages and environmental factors.

ASM does not own the copyrights to Supplemental Material that may be linked to, or accessed through, an article. The authors have granted ASM a non-exclusive, world-wide license to publish the Supplemental Material files. Please contact the corresponding author directly for reuse.
