# Supplementary material for: Novel phage infecting the Roseobacter CHUG lineage reveals a diverse and globally distributed phage family
Source: mSphere. 2024 Jun 27;9(7):e00458-24. doi: 10.1128/msphere.00458-24 (PMC11288001; doi:10.1128/msphere.00458-24)
Supplement: Supplemental figures — Fig. S1 to S6 [file msphere.00458-24-s0001.docx]

**Supplementary information for**

**Novel phage infecting the *Roseobacter* CHUG lineage reveals a diverse and globally distributed phage family**

Wu Zuqing, Guo Luyuan, Wu Ying, Yang Mingyu, Du Sen, Shao Jiabin, Zhang Zefeng *, Zhao Yanlin*

Fujian Provincial Key Laboratory of Agroecological Processing and Safety Monitoring, College of JunCao Sciences and ecology, Fujian Agriculture and Forestry University, Fuzhou, China.

**SUPPLEMENTAL LEGENDS**

**FIG S1** Phylogenetic tree based on the 16S rRNA gene sequences of *Roseobacter*. Circle size in the phylogeny indicates nodes with different bootstrap values. The FZCC0198 is labeled as red.

**FIG S2** Heatmap of the average amino acid identity (AAI) and shared genes percentage between CRP-810-type phages. The phylogenic core genes of CRP-810-type phages are shown on the left. The upper and lower triangles of the heatmap represent the shared gene percentage and AAI for each CRP-810-type phage, respectively.

**FIG S3** Distribution of different functional modules in the subgroups. Genes that were not detected in the genomes were labeled white. Different subgroups were labeled as different colors.

**FIG S4** The result of PCR amplification of the attachment sites attL and attR of CRP-810.

**FIG S5** FIG S5 The number of marine viromes of the widely distributed CRP-810-type phages in different marine biomes. Polar, westerlies and trades were colored blue, orange, and red, respectively.

**FIG S6** Comparison of relative abundance between members with higher G + C content (> 45) and low G + C content (< 45). The significance of pairwise comparisons calculated using the two-tailed Mann–Whitney U test was shown, with the asterisk corresponding to the pvalue (* pvalue < 0.05, **pvalue < 0.01, ***pvalue < 0.001).

**TABLE S1.** Genome annotation of CRP-810.

**TABLE S2.** General features of the CRP-810-type genomes.

**TABLE S3.** List of protein orthologous groups identified from the CRP-810-type genomes.

**TABLE S4.** Distance between CRP-810-type phages and other related known phages based on the gene contents

**TABLE S5.** Potential host of the CRP-810-type phages predicted by Rafah.

**TABLE S6.** Correlation between the CRP-810-type phages and environmental factors.


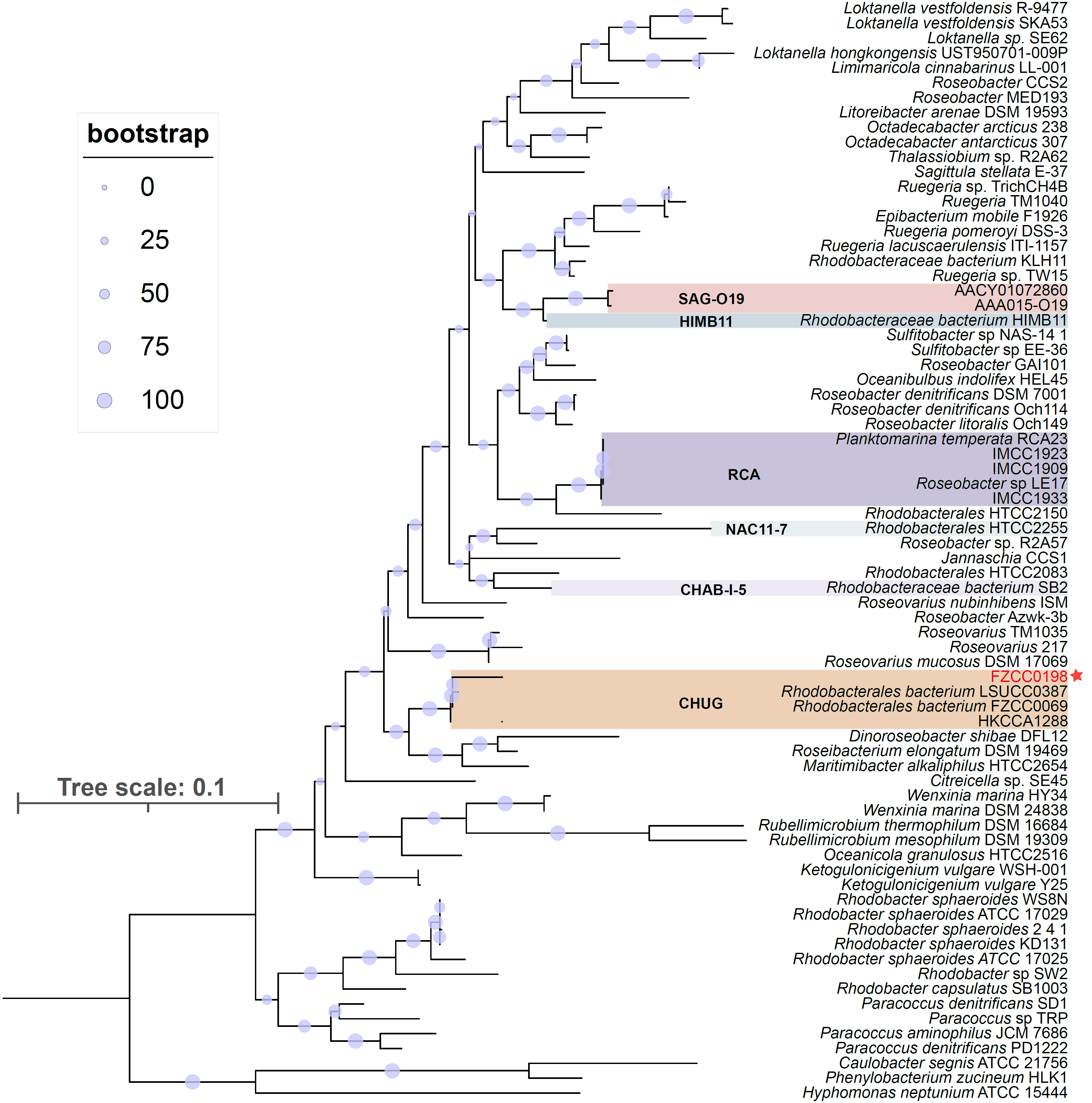


**FIG S1** Phylogenetic tree based on the 16S rRNA gene sequences of *Roseobacter*. Circle size in the phylogeny indicates nodes with different bootstrap values. The FZCC0198 is labeled as red.


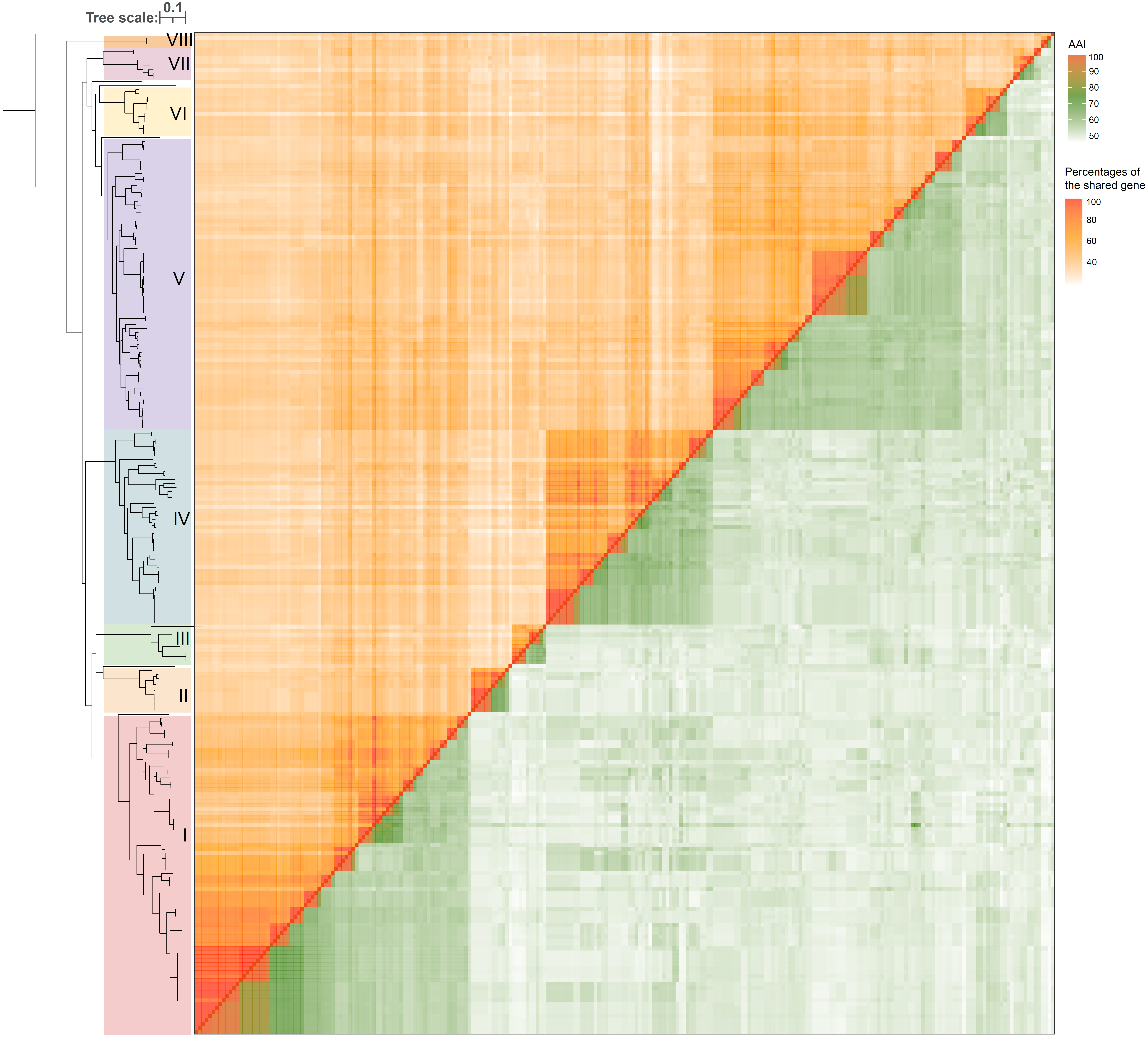


**FIG S2** Heatmap of the average amino acid identity (AAI) and shared genes percentage between CRP-810-type phages. The phylogenic core genes of CRP-810-type phages are shown on the left. The upper and lower triangles of the heatmap represent the shared gene percentage and AAI for each CRP-810-type phage, respectively.


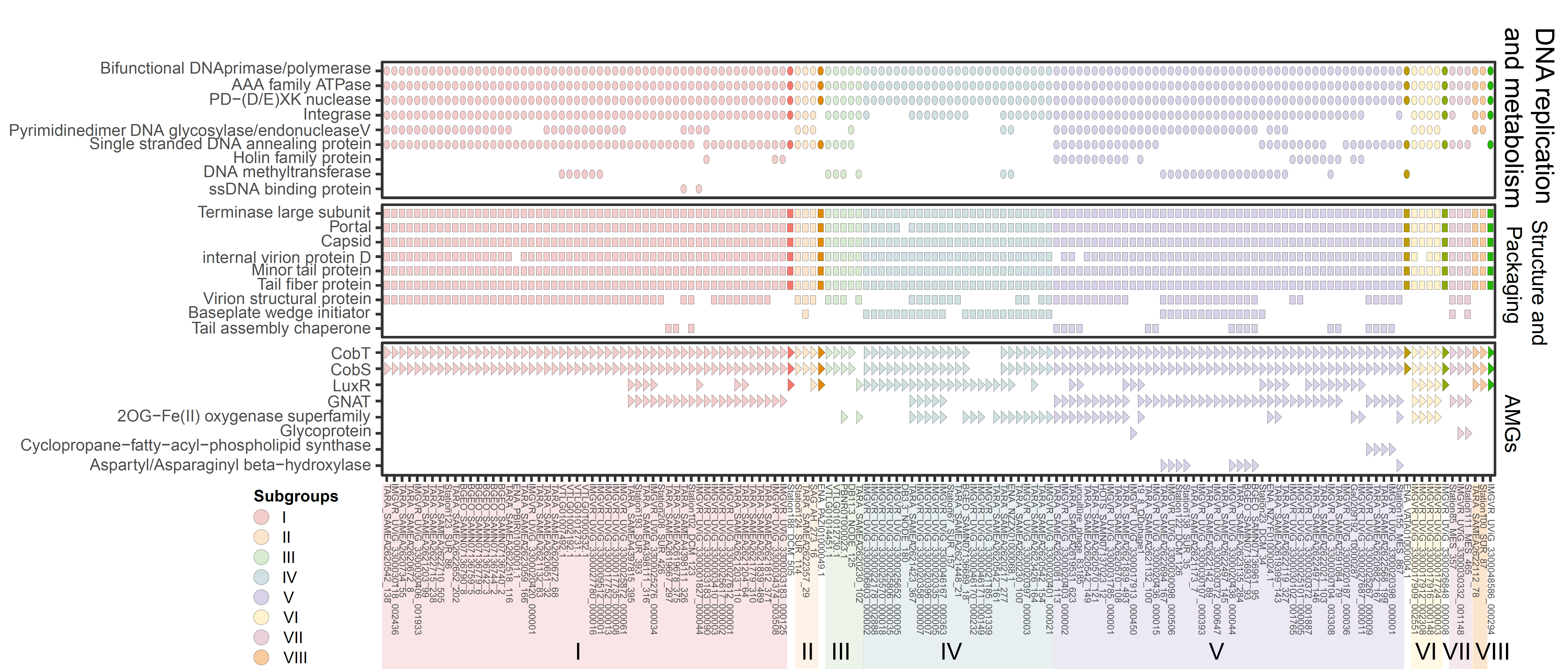


**FIG S3** Distribution of different functional modules in the subgroups. Genes that were not detected in the genomes were labeled white. Different subgroups were labeled as different colors.

**FIG S4** The result of PCR amplification of the attachment sites attL and attR of CRP-810

**FIG S5** The number of marine viromes of the widely distributed CRP-810-type phages in different marine biomes. Polar, westerlies and trades were colored blue, orange, and red, respectively.


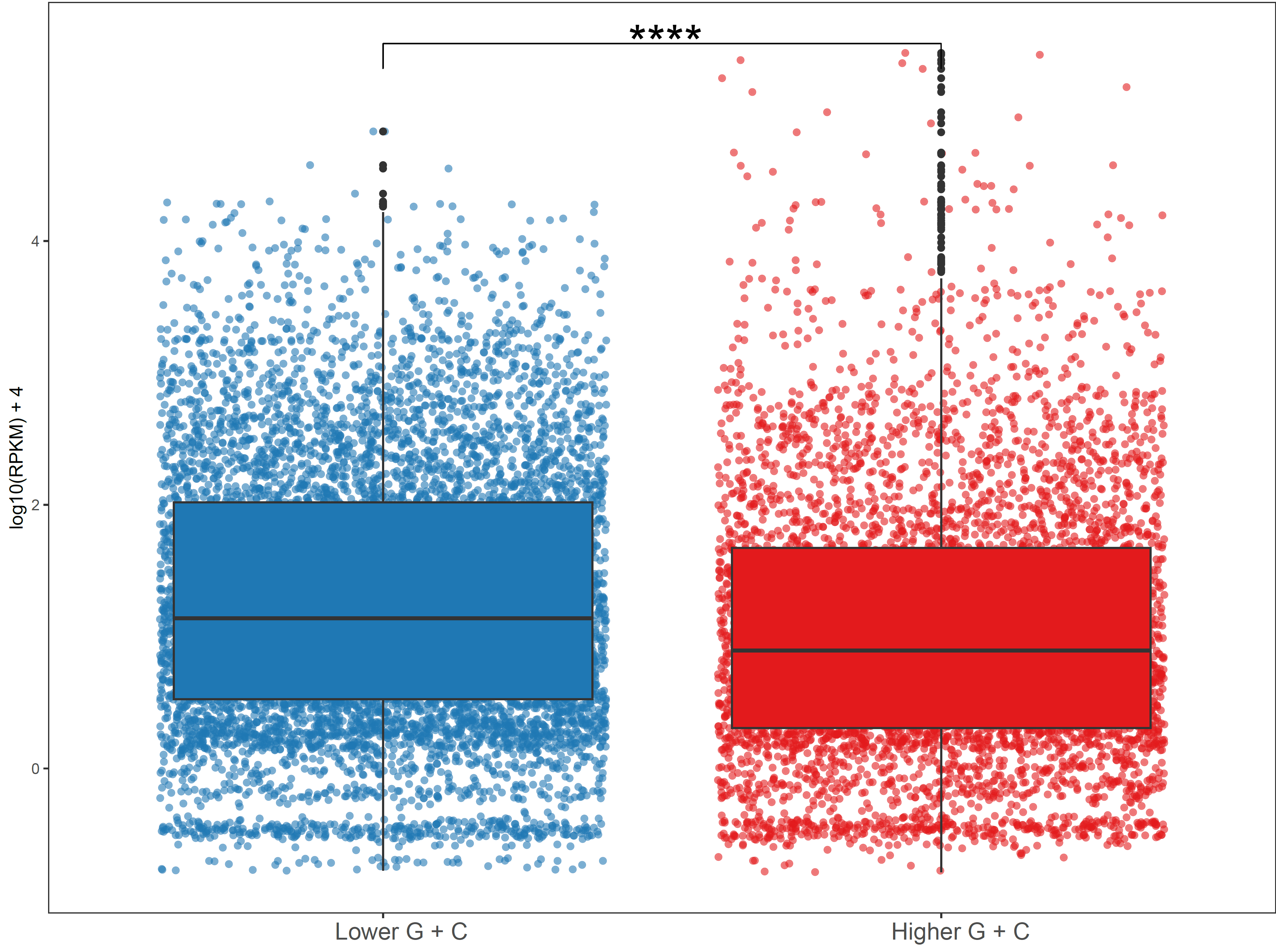


**FIG S6** Comparison of relative abundance between members with higher G + C content (> 45) and low G + C content (< 45). The significance of pairwise comparisons calculated using the two-tailed Mann–Whitney U test was shown, with the asterisk corresponding to the pvalue (* pvalue < 0.05, **pvalue < 0.01, ***pvalue < 0.001).
